# 4155 THE ROLE OF PERIODONTAL DISEASE IN CORONARY ARTERY DISEASE IN A HISPANIC POPULATION

**DOI:** 10.1017/cts.2020.152

**Published:** 2020-07-29

**Authors:** Pablo I Altieri, Kiara Didriksen, Pablo Altieri, Hector L. Banchs, Nelson Escobales

**Affiliations:** 1 University of Puerto Rico-Medical Sciences Campus; 2 University of Puerto Rico, Medical Sciences Campus

## Abstract

OBJECTIVES/GOALS: The purpose of this report is to describe the role of Periodontal Disease (PD) in Coronary Artery Disease (CAD) in a Hispanic country. METHODS/STUDY POPULATION: Literature and Puerto Rican experience was reviewed and will be discussed. RESULTS/ANTICIPATED RESULTS: PD produces inflammatory disease by bacterial infection in the gingiva. This factor PD activates an inflammatory process affecting the CAD cascade inducing myocites, endothelial cells activation and cytokines. The incidence of gingival disease in the Puerto Rican population (P) is around 50%; of this group 80% will develop periodontal disease. Including this factor and diabetes mellitus Type 2, still the incidence of CAD is 20-30% less than the U.S.A. DISCUSSION/SIGNIFICANCE OF IMPACT : CAD is a systemic disease related to genetic factors and inflammation. PD is related to an inflammatory process, which will activate the CAD process, producing tissue infarcts. The daily use of resolving or liquid Omega 3 in the gingival tissue is useful in the prevention of gingival and periodontal disease. CONFLICT OF INTEREST DESCRIPTION: All authors have no relationship with any industry or financial associations in connection with the submitted abstract.

